# Recent findings on chimeric antigen receptor (CAR)-engineered immune cell therapy in solid tumors and hematological malignancies

**DOI:** 10.1186/s13287-022-03163-w

**Published:** 2022-09-24

**Authors:** Ali Keshavarz, Ali Salehi, Setareh Khosravi, Yasaman Shariati, Navid Nasrabadi, Mohammad Saeed Kahrizi, Sairan Maghsoodi, Amirhossein Mardi, Ramyar Azizi, Samira Jamali, Farnoush Fotovat

**Affiliations:** 1 Department of Hematology and Blood Banking, School of Allied Medical Sciences, Shahid Beheshti University of Medical Sciences, Tehran, Iran; 2 Department of Oral and Maxillofacial Radiology, School of Dentistry, Islamic Azad University,, Isfahan (Khorasgan) Branch, Isfahan, Iran; 3 Department of Orthodontics, School of Dentistry, Alborz University of Medical Sciences, Karaj, Iran; 4 Department of General Surgery, School of Medicine, Arak University of Medical Sciences, Arak, Iran; 5 Department of Endodontics, School of Dentistry, Birjand University of Medical Sciences, Birjand, Iran; 6 Alborz University of Medical Sciences, Karaj, Iran; 7 Department of Paramedical, Kurdistan University of Medical Sciences, Sanandaj, Iran; 8 Department of Immunology, School of Medicine, Shahid Beheshti University of Medical Sciences, Tehran, Iran; 9 Department of Immunology, Faculty of Medicine, Tabriz University of Medical Sciences, Tabriz, Iran; 10 Department of Endodontics, College of Stomatology, Stomatological Hospital, Xi’an Jiaotong University, Shaanxi, People’s Republic of China; 11 Department of Prosthodontics, School of Dentistry, Hamadan University of Medical Sciences, Hamadan, Iran

**Keywords:** Immunotherapy, Chimeric antigen receptors, CAR T-cell, CAR-NK cell, CAR-M solid tumors, Hematological malignancies

## Abstract

Advancements in adoptive cell therapy over the last four decades have revealed various new therapeutic strategies, such as chimeric antigen receptors (CARs), which are dedicated immune cells that are engineered and administered to eliminate cancer cells. In this context, CAR T-cells have shown significant promise in the treatment of hematological malignancies. However, many obstacles limit the efficacy of CAR T-cell therapy in both solid tumors and hematological malignancies. Consequently, CAR-NK and CAR-M cell therapies have recently emerged as novel therapeutic options for addressing the challenges associated with CAR T-cell therapies. Currently, many CAR immune cell trials are underway in various human malignancies around the world to improve antitumor activity and reduce the toxicity of CAR immune cell therapy. This review will describe the comprehensive literature of recent findings on CAR immune cell therapy in a wide range of human malignancies, as well as the challenges that have emerged in recent years.

## Introduction

**Fig. 1:**
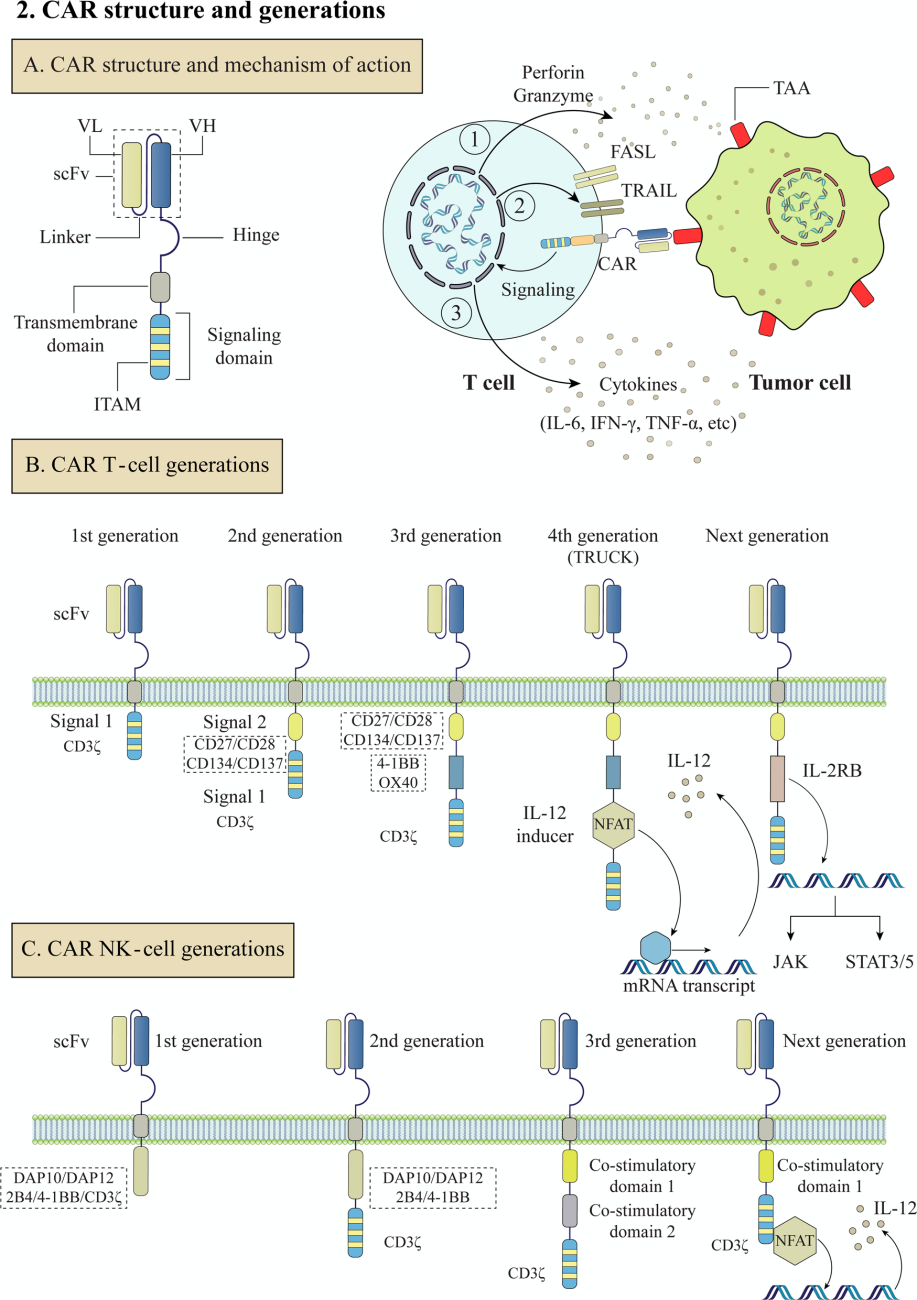
Chimeric antigen receptors' structure and generations. **A** CAR structure and mechanism of action. **B** CAR T-cell generations. **C** CAR NK-cell generations. Abbreviations: CAR (Chimeric antigen receptor), TAAs (tumor-associated antigens), NFAT (Nuclear factor of activated T-cells)

Malignant tumors are a primary global health concern, with increasing annual incidence and a high mortality rate. Surgery, chemotherapy, and radiation were the sole options for treating malignant tumors for decades [1]. In recent years, immune-based therapies such as chimeric antigen receptor (CAR)-modified immune cells have emerged as promising alternative treatments. Typically, the immune system eliminates potentially malignant cells, but cancer cells can acquire specific mutations that allow them to evade these mechanisms. Cancer immunotherapies aim to support or boost the patient's immune system so that cancer cells can be effectively eradicated [2]. One approach is to genetically modify immune cells, mainly T-cells and, more recently, natural killer (NK) cells and macrophages to express CARs. CAR expression on immune cells enables them to specifically target cancer cells by recognizing tumor-associated antigens (TAAs) [3]. Classical CARs are made up of an antigen recognition extracellular domain mainly derived from a monoclonal antibody fragment linked to intracellular T-cell receptor (TCR) complex binding domains [4] (Fig. 1). CAR binding to cell surface antigens is independent of the major histocompatibility complex (MHC) receptor, resulting in robust T-cell activation and potent antitumor responses [5]. CAR T-cells have demonstrated remarkable results in the treatment of relapsed or refractory hematological malignancies, culminating in the approval of six drugs by the Food and Drug Administration (FDA) between 2017 and the beginning of 2021 [6]. However, there are significant limitations to CAR immune cell therapy that must be addressed, such as life-threatening toxicities, diminished efficacy against solid tumors, limited durability, antigen escape, poor infiltration into the tumor, and the immunosuppressive features of the microenvironment [7]. On the other hand, many approaches, such as combining CAR immune cell therapy with other anticancer therapies or employing novel CAR engineering strategies, have been proposed to expand clinical efficacy, improve antitumor potency, and limit toxicities [4, 7]. In this review, we discuss ongoing innovations in CAR immune cell engineering to enhance clinical efficacy in solid tumors and hematological malignancies, as well as limitations of CAR immune cell therapy.

## Immune cells dysfunctions in the tumor microenvironment

**Fig. 2:**
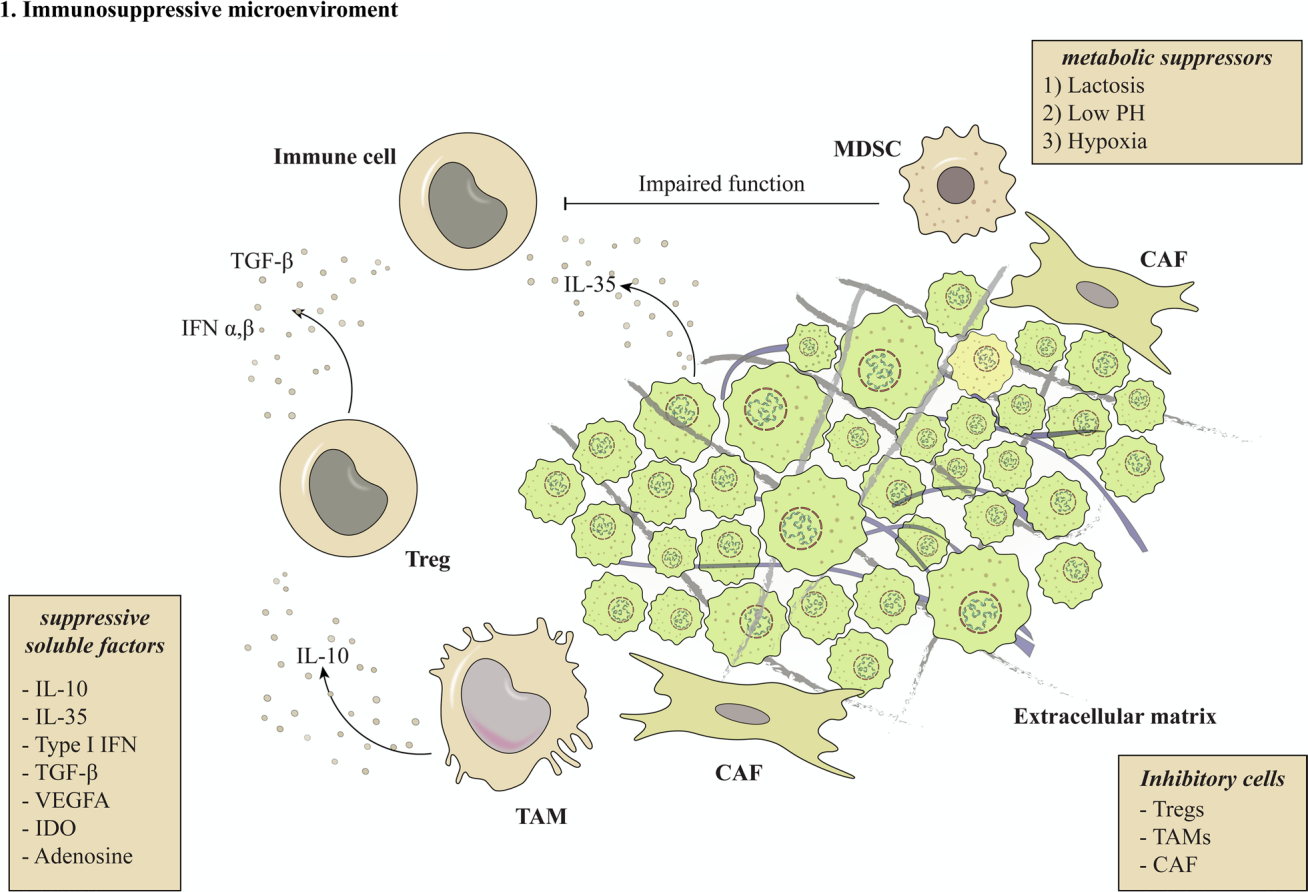
Tumor immunosuppressive microenvironment. The TME plays an important role in immune tolerance. The aspects of the complex TME that can sustain tumor growth, promote immune escape, and enhance immunosuppressive features are hypoxia, hypoglucosis, lactosis, acidity, and nutrient deprivation. Furthermore, changes in signal transduction molecules, the loss of tumor-specific antigens, stimulation of the inhibiting receptor CTLA-4 on T-cells, and some soluble molecules (IL-10, IL-35, type I IFNs, IDO, adenosine, VEGF-A, and TGF-) secreted by tumor cells or non-tumor cells in the TME all contribute to immune cell dysfunction. Moreover, the TME contains immunosuppressive cells (Tregs, MDSCs, TAMs, and CAFs) which contribute to immune cell dysfunction. Abbreviations: TME (tumor microenvironment), TSAs (tumor-specific antigens), CTLA-4 (cytotoxic T-lymphocyteassociated antigen 4), IFNs (interferons), IDO (Indoleamine 2,3-Dioxygenase), VEGF-A (vascular endothelial growth factor A), TGF-β, transforming growth factor-beta, Tregs (Regulatory T-cells), MDSCs (myeloid-derived suppressor cells), TAMs (tumor-associated macrophages), CAFs (cancer-associated fibroblasts)

The immune system has a crucial role in cancer growth control. Cancers, on the other hand, evolve to avoid detection by the immune system. Cancer growth and progression are a result of immune tolerance and active immune cell suppression [8]. The physiologic tumor microenvironment (TME) is a significant contributor to immune tolerance. Hypoxia, hypoglucosis, lactosis, acidity, and nutrient deprivation are the aspects of the complicated TME that can sustain tumor growth, promote immune escape, and enhance immunosuppressive features. Moreover, changes in the signal transduction molecules, loss of tumor-specific antigens (TSAs), stimulation of the inhibiting receptor cytotoxic T-lymphocyte-associated antigen 4 (CTLA-4) on T-cells as well as some soluble molecules (interleukin (IL)-10, type I interferons (IFNs), Indoleamine 2,3-Dioxygenase (IDO), adenosine, vascular endothelial growth factor A (VEGF-A), transforming growth factor-beta (TGF-β), and IL-35) secreted by tumor cells or non-tumor cells in the TME mediate immune cell dysfunction [9]. In addition, the TME contains several immunosuppressive cells that contribute to immune cell dysfunction. Regulatory T-cells (Tregs), myeloid-derived suppressor cells (MDSCs), tumor-associated macrophages (TAMs), and cancer-associated fibroblasts are examples of inhibitory cells [10, 11, 12] (Fig. 2). Immune-related therapies have been established to reverse dysfunctional immune cells and restore antitumor immunity. As a result, cancer patients gained many hopes for this approach. Currently, immunotherapy focuses on CAR-expressing immune cells in patients with various solid tumors and hematological malignancies [10].

## CAR structure and CAR-modified immune cells generation process

CAR-based genetically modified immune cells express synthetic receptors that efficiently redirect immune cells to the surface antigens on the tumors for tumor eradication via immune cell cytotoxicity. The typical structure of a CAR molecule consists of an extracellular antigen recognition domain (ectodomain), an extracellular hinge domain, a transmembrane domain, and one or more intracellular signaling endodomains [13, 14] (Fig. 1)**.** The ectodomain is known as the antigen recognition region and comprises a single-chain variable fragment (scFv) that targets a specific TAA. CAR T-cells' ectodomain (scFv) can recognize TAA on tumor cells without the need for classical antigen processing and presentation by the MHC, which is one of the main features of CAR T-cells. The hinge component (spacer) is responsible for connecting the ectodomain to the transmembrane domain. The transmembrane domain, hydrophobic alpha-helix, extends across the membrane between the spacer and the signaling end domains. [15, 16, 17]. The endodomain (signaling domain) is the functional component of CAR immune cells that controls their activation, proliferation, and survival. The CAR endodomain delivers costimulatory signals to immune cells in response to antigen recognition by the ectodomain, allowing them to initiate their cytotoxic function [17] (Fig. 1).

Four generations of CAR T-cells were constructed based on the endodomain structure [18]. The first generation of CARs had scFv attached to a single intracellular signaling domain, CD3. For efficient T-cell activation, two distinct signals are required. The interaction of the foreign peptide presented by the MHC complex with the TCR expressed on T-cells provides signal 1, and the interaction of costimulatory B7 molecules [B7.1 (CD80) or B7.2 (CD86)] expressed on the antigen-presenting cells with the co-receptor protein CD28 expressed on the T-cells provides signal 2 [19]. However, the first generation of CARs structure could only provide signal 1. Hence, T-cells are unable to carry out their efficient cytotoxic activity if costimulatory signal 2 is missing. As a result, the first CAR T-cell generations were ineffective, with low proliferation and high apoptotic potential [20]. To improve the limitations of the first CAR generation, in the second generation of CARs, two intracellular signaling domains (such as CD28 or 4–1BB) were introduced to the cytoplasmic tail of CARs [21]. In the third generation of CARs, Three costimulatory domains were introduced to improve T-cell activation signals and modify T-cell survival, produce more cytokines, and have a better antitumor cytotoxic effect [22, 23]. Finally, by adding a cytokine inducer to the base of a second-generation or the third-generation construct, the fourth generation of CAR T-cells, also known as T-cells redirected for universal cytokine killing (TRUCKs), was established [24, 25]. Likewise, next-generation CAR T-cells are currently underway. The new generation, already named the fifth generation, is based on the second-generation CARs with the addition of an IL-2 receptor *β* (IL-2R β) fragment. By mRNA transcription, the IL-2R β fragment can induce the production of Janus kinases (JAKs) and signal transducer and activator of transcription (STAT)-3/5 [18].

Additionally, other immune cells with antitumor function, like NK cells, demonstrate a promising potential to replace T-cells and for utilized in universal immune cell therapy, so researchers have focused on developing CAR-NK cells [26]. Although CAR constructs optimized for T-cell signaling were employed in early preclinical investigations of CAR-NK cells and some key costimulatory domains used in CAR design, such as 4–1BB, are shared by NK cells and T-cells, the significance of other costimulatory molecules in NK cells, such as CD28, is unclear [27]. This prompted several researchers to investigate costimulatory domains with higher specificity for NK cell signalings, such as DAP10, DAP12, or 2B4 (Fig. 1).

## Immune cells sources

The majority of current CAR T-cells are made of harvested T-cells from patients. However, a high burden of tumors and a lack of T-cell population, particularly in patients with T-cell malignancies or those who have received chemotherapies, have made it challenging to obtain a sufficient quantity of competent T-cells by apheresis. In addition, allogeneic CAR T-cell therapy has been demonstrated to be effective in patients with suitable donors, and speculation that allo-hematopoietic stem-cell transplantation (HSCT) causes severe graft-versus-host disease (GVHD). Nevertheless, allo-CAR T-cell treatment is costly and time-consuming even for patients who meet the donor-matching requirements (Table 1) [28, 29].

**Table 1:** CAR immune cell sources

CAR immune cell	Sources	Explanation	References
CAR T-cell	Autologous CAR T-cell	•Harvest T-cells from patients •Low risk for GVHD •Difficult to obtain a sufficient quantity of T-cells by apheresis in patients with T-cell malignancies or malignancies that receive chemotherapies •Patients with rapidly progressing infections or cancers may not survive for several weeks needed to produce CAR T-cells •Expensive	[28, 30]
	Allogeneic CAR T-cell	•Need for suitable donors •Causes severe GVHD •Allogeneic cells can be prepared and stored for future use so that there is a shorter waiting period vs. auto-CARs for infusion into the patient •Expensive	[28, 29, 30]
CAR-NK cells	NK-92 cell line	•Easy to expand in vitro •Source of limitless number of CAR-NK cells •As the engineered NK-92 cells are of malignant origin, the cells must be irradiated. Irradiation shortens the survival of CAR-NK92 cells in the peripheral blood of the recipient •They are naturally deprived of the CD16 domain, and are hence unable to trigger ADCC	[31, 32]
	Peripheral blood mononuclear cells (PBMCs)	•Mature NK cells can be easily harvested •Relatively few cells can be obtained from each donation •90% of the NK cell population in PB unfortunately do not expand easily in vitro •The cells obtained from PB respond more effectively and persist in circulation for longer than NKs from other sources	[32, 33, 34]
	Umbilical cord blood (UCB)	•NK cells constitute about 30% of the lymphocytes in UCB •Inferior cytotoxic capabilities compared to PB-derived NK cells •Greater potential to expand than PB-derived NK cells	[34, 35]
	Induced progenitor stem cells (iPSC)	•Harvest from the mobilized PB or from UCB •The major virtue of iPSC-derived CAR-NK cells is the potential to produce large numbers of homogeneous CAR-NK cells from one iPSC •This technology generates cells with an immature, less cytotoxic phenotype, similar to UCB-derived NK cells	[36, 37]

GVHD, graft-versus-host disease; ADCC, antibody-dependent cell cytotoxicity; PB, peripheral blood

Unlike T-cells, NK cells have a lower risk of GVHD, opening the path to producing “off-the-shelf” allogeneic cell therapy agents that can be prepared ahead of time and made available to a large number of patients on demand [38]. Clinical-grade NK cells can currently be produced in large quantities using a variety of sources (Table 1) [39].

## Similarities and differences between CAR T-cells and CAR-NK cells

With the dramatic success of CAR-engineered T-cells in the treatment of hematological cancers, there are flaws in CAR T-cell therapy's broad therapeutic application. On the other hand, CD8^+^ cytotoxic T-cells and NK cells are two types of immune cells that use similar cytotoxic mechanisms to kill target cells [40]. As a result, there is a growing interest to develop CAR-engineered NK cells as a low-cost cancer therapy [41]. CAR-NK cells may have some significant advantages over CAR T-cells [27, 42]. Currently, CAR T-cells are made by collecting blood from the patients, creating the CAR T-cells, and then administering them to the patient. This takes two main items to consider. One is the cost, and second, some patients with particularly aggressive illnesses may not have the four or six weeks required for CAR engineering. Nonetheless, with CAR-NK cells, we can have an off-the-shelf product so that the patient comes into your clinic and you have these cells frozen, sitting in a biobank, and you can thaw and infuse, making the strategy lot more appealing and also cheaper [7, 43]. In addition, CAR-NK cells are safer than CAR T-cells because the cytokines secreted by activated NK cells (e.g., IFN-γ and granulocyte–macrophage colony-stimulating factor (GM-CSF) are safer and typically eliminate the risk of cytokine storm and extreme neurotoxicity caused by pro-inflammatory cytokines (tumor necrosis factor-alpha (TNF-*α*), IL-1, and IL-6) released by CAR T-cells [44], and also allogeneic CAR-NKs decrease the risk of GVHD because they are not restricted to MHC [45]. Moreover, CAR-NK cells may have multiple mechanisms for cytotoxic activity because they can recognize and kill targets via engineered killing capacity as well as their intrinsic killing capacity via natural cytotoxicity receptors [46, 47]. Furthermore, CAR-expressing NK cells enable the eradication of heterogeneous malignancies in which some malignant cells lack CAR targeted specific antigens [48]. Indeed, mature NK cells have a shorter lifespan in the bloodstream, which reduces the risk of long-term cell deficiency caused by cellular memory responses and on-target/off-tumor effects [43]. Furthermore, because of the low risk of alloreactivity and GVHD, allogenic CAR-NK cells could potentially be obtained from various sources, including PB, UCB, iPSCs, hESC, and NK-92 cell lines [49, 50]. Despite many advantages of NK cells, the utilization of CAR-NK cells in clinical trials faces some challenges. The current CARs used in NK cells have a structure that causes the first reluctance. The epitopes binding to the current CAR's position, as well as their space from the CAR-NK cell's surface, reduce the ability of these cells to bind antigens and stimulate CAR-NK cells [51]. NK cells are also more susceptible to freezing and thawing, which reduces their antitumor capacity and survival rate [52]. Indeed, because infused CAR cells do not persist in the absence of cytokine assistance, exogenous cytokines must be given in a certain order for infused NK cells to survive and proliferate in vivo. Exogenous cytokines, on the other hand, might have adverse consequences such as systemic toxicity [53, 54]. The final issue stems from the lack of an effective gene transfer strategy in NK cells [55] Retro- and lentiviral vectors were used in most studies on viral transduction of NK cells, but their use has been limited. This could be due to innate NK cell properties like pattern recognition receptors that identify foreign genomic material, which is probably associated with triggering NK cell apoptosis after viral transduction. In addition, electroporation or lipofection can be used to transfer genes via transfection. Transfection of NK cells is found to be correlated with lower levels of apoptosis and transgene transport efficacy that is fully independent of cellular division as compared to viral transduction [56] (Table 2). Finally, it is essential to note that unlike CAR T-cells, CAR-NK cell studies are still mostly preclinical, but there is a lot of promise from CAR-NK cell-based studies. Therefore, we need to wait and see how well they act in the clinic.

**Table 2:** Comparison between CAR T-cells and CAR-NK cells

Parameter	CAR T-cells	CAR-NK cells
Sources	Mainly autologous T-cells	Variety of sources, including PB, UCB, HPCs, hESC, iPSCs, and cell lines
Safety	May causes GVHD and cytokine storm	Safer, reduce the risk of cytokine storm and GVHD
Cytotoxic mechanism	CAR-restricted cytotoxicity	Multiple mechanisms for cytotoxic activity, CAR killing capacity as well intrinsic killing capacity via natural cytotoxicity receptors
HLA restriction	No HLA restriction	No HLA restriction
Transduction	Higher transduction efficacy	Low transduction efficacy
Persistence	Better persistence	Low persistence, need to exogenous cytokines
Stability	Less susceptible to freezing and thawing	More susceptible to freezing and thawing
Life span	Longer	Shorter
Cost of production	Expensive	Cheaper
Construction to injection period	Longer period of time (4–6 weeks)	Short period time, off-the-shelf

PB, peripheral blood; UCB, umbilical Cord blood; HPCs, hematopoietic progenitor cells; hESC, human embryonic stem cell; iPSCs, induced pluripotent stem cells; GVHD, Graft-versus-host disease; HLA, human leukocyte antigen

## CAR-modified immune cells in hematological malignancies

### Lymphoma

For lymphoma, the most common therapies were chemotherapy regimens and monoclonal antibodies. Although these agents were successful to some extent, some patients' diseases deteriorated even after primary and secondary therapies [57]. However, there is a novel and effective alternative treatment for patients who have failed multiple lines of therapy [58]. Therefore, immune cell therapy with CAR T-cell has transformed the treatment of lymphomas, particularly for aggressive B-cell lymphomas. As a result, the FDA has licensed four anti-CD19 CAR T-cells for aggressive B-cell lymphoma (axicabtagene ciloleucel, tisagenlecleucel, brexucabtagene autoleucel and lisocabtagene ciloleucel). Although these products have different CAR designs, costimulatory domains, manufacturing processes, doses, and pivotal trial eligibility criteria, all four CAR T-cells generate long-term remissions in 33–40% of treated patients [59]. Axicabtagene ciloleucel (axi-cel) is an anti-CD19 CAR T agent delivered via retrovirus using a CD28 costimulatory signal. The ZUMA-1 trial was a landmark study that examined axi-cel and resulted in the first FDA approval of CAR T-cell therapy to treat large B-cell lymphomas in 2017 [60, 61]. Tisagenlecleucel (Kymriah) is another anti-CD19 CAR T agent that uses lentivirus-based vector delivery with a 4-1BB costimulatory signal. The JULIET trial (NCT02445248) was a global phase II trial that demonstrated tisagenlecleucel to have high response rates and a long duration of action in adults with refractory or relapsed or diffuse large B-cell lymphoma (DLBCL) [62]. Brexucabtagene autoleucel (Tecartus) is another anti-CD19 CAR T that was approved in 2020 for the mantle cell lymphoma (MCL) treatment and recently for ALL based on ZUMA-2 (NCT02601313) and ZUNA-3 (NCT02614066) trials [63, 64]. The last FDA-approved agent is Lisocabtagene maraleucel (liso-cel; JCAR017), a CD19-directed 4-1BB CAR T product for the treatment of adult patients with refractory or relapsed large B-cell lymphoma [65]. Besides CD19, some other surface biomarkers are essential. CD20, a transmembrane protein found in more than 90% of B-cell lymphomas, is a well-known target for non-Hodgkin lymphoma (NHL) treatment [66]. Several studies are currently using first-generation anti-CD20 CAR T-cell therapy (NCT04160195, NCT03576807, NCT03019055). Another possible target is CD30. Classical Hodgkin lymphoma (HL), anaplastic large cell lymphoma, DLBCL, primary mediastinal B-cell lymphoma, and peripheral T-cell lymphoma all express CD30 [67, 68]. Furthermore, the use of Epstein-Barr virus (EBV)-specific CAR T-cells that recognize and kill EBV-infected cells may protect against the recurrence of EBV-related B-cell NHLs such as BL and DLBCL. The κ or λ light immunoglobulin chain expressed in some mature malignant B-cells and anti-κ/λ CAR T-cells are also potential strategies for the treatment of B-cell lymphoma [67].

CAR-NK cells have also been investigated in the treatment of lymphoma (Table 3). The safety and efficacy of anti-CD19 and anti-CD20 CAR-NK cells against B-cell malignancies were primarily investigated in preclinical studies with CAR-NK cells, but outcomes with first-generation CARs were modest [69]. Liu et al. revealed that genetically modified NK cells from cord blood using a retroviral vector expressing a fourth-generation CAR vector (iC9.CAR19.CD28ζ-IL-15) exhibited remarkable antitumor activity in xenograft mouse models of Raji tumor activity [70]. Chu et al. also used mRNA nucleofection to genetically modify peripheral blood NK cells from healthy donors to express anti-CD20 CAR. In preclinical models, this approach resulted in 67% CAR expression and significant in vitro and in vivo efficacy against Burkitt lymphoma [71]. Chen et al. found that third-generation anti-CD5 CAR Transduced NK-92 cells with CD28 and 4-1BB costimulatory domains have potent in vitro cytotoxicity against primary patient-derived -cell acute lymphoblastic leukemia (T-ALL), T-cell lymphoma, and Sezary cells, as well as enhanced survival in a Jurkat lymphoma xenograft mouse model [72].

**Table 3:** Summary of CAR-NK cell clinical trials

Type of malignancy	CAR-NK Cell product	Phase stage	Status	Last update posted	Identifier
B-cell NHL, ALL, CLL, WM	Allogeneic CAR-NK cells targeting CD19	Phase 1	Recruiting	November 17, 2021	NCT05020678
Multiple myeloma	Anti-BCMA CAR-NK Cells	Early Phase 1	Recruiting	November 23, 2021	NCT05008536
AML/MDS	Allogeneic CAR-NK targeting NKG2D	Phase 1	Recruiting	May 17, 2021	NCT04623944
AML	anti-CD33 CAR-NK cells	Phase 1	Not yet recruiting	August 17, 2021	NCT05008575
B-cell NHL, ALL, CLL	CAR-NK-CD19 Cells	Phase 1	Recruiting	April 8, 2021	NCT04796675
Hematological malignancy	CD5 CAR-engineered IL15-transduced cord blood-derived NK cells	Phase 1 Phase 2	Not yet recruiting	November 8, 2021	NCT05110742
B-cell Lymphoma, ALL, CLL	CAR-NK-CD19 Cells	Phase 1	Recruiting	March 15, 2021	NCT04796688
Multiple Myeloma	BCMA CAR-NK 92 cells	Phase 1 Phase 2	Recruiting	May 7, 2019	NCT03940833
NHL	Anti-CD19 CAR-NK	Early Phase 1	Not yet recruiting	November 20, 2020	NCT04639739
B-cell NHL	Anti-CD19 CAR-NK	Phase 1	Recruiting	May 14, 2021	NCT04887012
B-Cell lymphoma, MDS, AML	CAR.70- Engineered IL15-transduced Cord Blood-derived NK Cells	Phase 1 Phase 2	Not yet recruiting	November 2, 2021	NCT05092451
B-cell NHL	CD19 CAR-NK cell therapy (TAK-007)	Phase 2	Not yet recruiting	November 11, 2021	NCT05020015
Malignant tumor	ROBO1 BiCAR-NK/T-cells	Phase 1 Phase 2	Recruiting	April 30, 2019	NCT03931720
Glioblastoma	HER2-specific, CAR-expressing NK-92 cells (NK-92/5.28. z)	Phase 1	Recruiting	September 25, 2020	NCT03383978
Prostate cancer	Anti-PSMA CAR-NK cells	Phase 1 Phase 2	Not yet recruiting	October 2, 2018	NCT03692663
Gastroesophageal junction cancers, Advanced HNSCC	PD-L1 CAR-NK Cells	Phase 2	Recruiting	November 23, 2021	NCT04847466
Pancreatic cancer	ROBO1 CAR-NK cells	Phase 1 Phase 2	Recruiting	May 8, 2019	NCT03941457
Solid tumors	ROBO1 CAR-NK cells	Phase 1 Phase 2	Recruiting	May 7, 2019	NCT03940820

HNSCC, Head and neck squamous cell carcinomas; NHL, Non-Hodgkin lymphoma; ALL, Acute lymphoblastic leukemia; CLL, Chronic lymphocytic leukemia; MW, Waldenstrom macroglobulinemia; AML, Acute myeloid leukemia; MDS, Myelodysplastic syndrome

### Leukemia

Although CAR T-cell therapy has been shown to be highly effective in treating ALL and that two CD19-CAR T-cell therapies (tisagenlecleucel and Brexucabtagene autoleucel) have been approved by the FDA for the treatment of refractory or relapsed ALL, relapsing ALL-blasts with down-regulated or abrogated CD19 expression remain a critical barrier to maintaining permanent remissions [73, 74]. As a result, backup antigens such as CD22 have received increased attention. In a recent clinical trial, CD22-CAR T-cells were shown to have the potential to mediate complete remissions in patients with relapsing CD19-negative blasts [75, 76]. Although evidence with CAR T-cells in chronic lymphocytic leukemia (CLL) therapy is limited, safety and efficacy outcomes are promising, implying that CAR T-cells might be employed in CLL patients with a dismal prognosis [77]. Although several target antigens, including CD20 [78], ROR1 [79], CD23 [80], and κ-light chain [81], were studied in CLL, the most promising clinical data, similar to ALL, was obtained using CD19-CAR T-cells [82]. Despite this, the overall complete remission rate for CD19-CAR T-cells in CLL was only 29% [82]. CLL patients' T-cells have been shown to acquire proliferative and metabolic dysfunctions, which may contribute to the lower efficacy of CAR T-cell therapy in CLL [83]. Unlike ALL and CLL, clinical knowledge on CAR T-cells in acute myeloid leukemia (AML) is still being developed. The main barrier to applying CAR T-cell therapy to myeloid malignancies is the lack of distinct target antigens that distinguish malignant cells from healthy progenitor cells [84]. A number of target antigens have been proposed for a long time, including CD123 [85], LeY antigen [86], folate receptor-β [87], and CD33 [88]. CAR T-cells targeting Lewis-Y on myeloid blasts provided proof-of-principle results for CAR T-cell efficacy in AML, but there was no report of long-term remission [89]. CD123 and CD33 have appeared as the premier target antigens getting the most attention in current clinical trials due to their universal presence in primary diagnosis and relapsed myeloid blasts [90, 91]. In a current trial, CAR T-cells targeting CD123, which is expressed on leukemia-initiating cells, were found to elicit full remissions (NCT02159495) [89]. FMS-like tyrosine kinase 3 (FLT3) is another promising target antigen that could be successfully exploited using FLT3-targeting CAR T-cells demonstrating potent reactivity against AML blasts expressing wild-type or FLT3 with internal tandem duplication (FLT3-ITD) [92].

A phase I clinical trial of CD33-CAR-NK cells reported the safety of CAR-NK cell therapy for relapsed and refractory AML patients [93]. Another study indicated that FLT3-CAR-NK cells might successfully target B-ALL [94]. Furthermore, CD19-CAR-NK-92 cells inhibited leukemia growth in a B-cell lymphoma mouse model [95]. On the other hand, AML blasts are extremely sensitive to NK cell-mediated toxicity as they express several of the ligands identified by NK cell activation receptors. As a result, CAR-NK cells may be able to overcome some of the hurdles associated with antigen evasion and tumor heterogeneity in AML through their innate ability to detect and target AML cells [27, 96]. Thus, attempts to develop CAR-NK cells against AML are now underway. A recent study showed that primary human NK cells with anti-CD123 and dual co-stimulation with CD28 and 4-1BB successfully destroyed AML cell lines and primary AML blasts in vitro [96]. In another investigation, Salman et al. demonstrated that injecting CD4-CAR-NK cells into a systemic AML implanted murine model may enormously boost anti-leukemic effects [97]. In addition, CAR-NK cell therapy may be effective in the treatment of T-cell-related leukemia. CD7-CAR-NK-92 demonstrated selective and potent antitumor activity against T-ALL cell lines and T-ALL xenograft mouse models, which could be mediated by increased granzyme B and IFN-γ secretion [98, 99].

### Myeloma

Even though CD19 CAR T-cells demonstrated remarkable results in treating certain B-cell malignancies, it was unable to effectively eliminate myeloma cells due to the lower expression of CD19 on the surface of myeloma cells [100, 101]. Therefore, alternative markers were considered, and B-cell maturation antigen (BCMA) received the most remarkable attention [102, 103]. BCMA, a tumor necrosis factor (TNF) receptor superfamily member that binds both B-cell activating factor and a proliferation-inducing ligand (APRIL), is widely and almost exclusively expressed on plasma cells and B-cells [104]. Early clinical trials with BCMA CAR T-cells in strongly pretreated individuals demonstrated a high response rate; however, relapses did occur [105]. As anti-BCMA trials were done and relapses were identified, a variety of markers were investigated as prospective targets, including APRIL [106], CD138 [107], kappa light chain [81], CS1 glycoprotein antigen (SLAMF7) [108], GPRC5D [109], CD38 [110], CD229 [111], Lewis-Y (NCT01716364), and NKG2D ligands [112]. Although the cohorts were small, treatment efficacy ranged from no response to 80%, with many complete remissions recorded [113]. In addition, CD19-targeted CAR T-cells used in combination with autologous stem-cell transplantation have shown efficacy in relapsed/refractory multiple myeloma [114].

Myeloma cells are also susceptible to NK cell-mediated death as they express various ligands for NK receptors [115]. Hence, there is much interest in creating CAR-NK cells that target myeloma antigens such as CS1 (CD319), CD138, and BCMA [69]. Therefore, we transduced NK-92 cells with a lentiviral vector expressing a first-generation anti-CD138 CAR or a second-generation anti-CS1 CAR.CD28. ζ demonstrated in vitro and in vivo activity against myeloma [116]. Similarly, an investigation into the potential therapeutic effects of CD38-specific nanobody-based CAR (Nb-CARs) revealed that Nb-CAR-NK-92 cells might induce cytotoxicity against primary patient-derived CD38-expressing myeloma cells [117].

## CAR-modified immune cells in solid tumors

CAR immune cell therapy has developed as an immunotherapy modality, and after achieving remarkable success in hematological malignancies, it is now being extensively studied in advanced solid tumors. However, unlike in hematological malignancies, CAR immune cell application in solid cancers has been limited by a number of challenges. Several methods have been developed to overcome these limitations, including localized delivery of CARs, genetic alterations, and combination therapy [118]. Overall, 22 targets are being studied in patients with solid tumors in ongoing or published clinical trials around the world (FR-α, MSLN, human epidermal growth factor receptor 2 (HER2), epidermal growth factor receptor (EGFR), carcinoembryonic antigen (CEA), MUC1, GD2, epithelial cell adhesion molecule (EpCAM), carbonic anhydrase IX (CAIX), L1-CAM, EGFRvIII, IL13Rα2, prostate-specific membrane antigen (PSMA), PSCA, fibroblast activation protein (FAP), CD133, c-MET, ephrin type-A receptor 2 (EphA2), Glypican-3 (GPC3), VEGFR-2, ROR1, MUC16) [119].

### Breast cancer

Triple-negative breast cancer (TNBC) is the complex and the most challenging subtype of breast cancer, with a very high recurrence rate and a poor prognosis, necessitating a substantial effort to identify novel treatment options [120]. The initial step to creating successful CAR therapies for TNBCs is the selection of relevant cell surface antigens. TSAs are ideal CAR targets since they contribute to cancer pathobiology and are shared by cancer patients. However, TSAs expressed in a high ratio of patients with cancer are limited [121]. Antigens that are now being targeted in breast cancer clinical research include HER2, mesothelin, CEA, CAIX, FR-α, CD171, GD2, EGFRvIII, FAP, and vascular endothelial growth factor receptor2 (VEGF-R2) [122, 123, 124]. Mucin 1 (MUC1) is expressed in more than 90% of breast cancers, making it the most relevant and significant antigen for breast cancer targeting. Furthermore, MUC1 is expressed on 95% of TNBC. Zhou et al. demonstrated that the destruction of TNBC tumors by TAB004-derived MUC28z CAR T-cells is effective in vitro and in vivo [125]. Because MUC1 is expressed in the majority of epithelial-derived solid tumors, such as breasts cancer subtypes, MUC28z CAR T-cells will most likely have extensive applicability for solid tumor targeting [125]. Breast cancer recurrence and, ultimately, survival are known to be influenced by HER-2 expression. HER-2-targeted therapies are now an essential part of the therapy of HER-2 overexpressing breast cancer [126]. Several HER-2-targeted CAR T-cell clinical trials are currently underway, including a phase I HER2-targeted dual switch CAR T-cell (BPX-603) in subjects with HER2-positive solid tumors (NCT04650451) and a phase 1 combination of binary oncolytic adenovirus in advanced HER2- positive solid cancers with HER2-specific autologous CAR VST (NCT03740256). Tchou et al. showed overexpression of mesothelin in 67% of TNBC samples, with minimal expression in other breast cancer subtypes and no expression in normal breast epithelial cells. Moreover, CAR T-cells specific for mesothelin showed cytotoxicity against TNBC cells [127]. TNBC immunotherapy using CAR T-cells is still in its early stages, but it has much promise. Target selection, CAR creation, preclinical and clinical testing, and techniques to improve safety and boost the CAR T-cell immunotherapy response are all part of the current work, which is critical to the success of immunotherapy in TNBC and other solid cancers [128].

On the other hand, breast cancer is a potent target for CAR-NK therapy [129]. An in vitro analysis revealed that ErbB2-CAR-NK-92 cells successfully eradicated ErbB2 + breast cancer cells [31]. Hu et al. showed that tissue factor (TF) targeted NK cells efficiently destroyed TNBC cells in vitro and a TNBC xenograft model [130]. Indeed, Liu et al. found that employing EGFR-CAR-NK cells accurately triggered in vitro lysis of TNBC cells and suppressed tumor development in a mouse model [131].

### Ovarian cancer

There are two main advantages of studying CAR T-cells for ovarian cancer (OC) treatment. First, OC cells express a wide range of TAAs [132]. Aside from CA125, the cancer cell expresses more than 60 TAAs. This increases the number of targets available for CAR T-cell design [133]. To date, TAAs found in OC include MUC16, HER2, hepatocyte growth factor receptor (c-Met), mesothelin, folate receptor alpha (FRα), cancer/testis antigen 1B, and cancer antigen 125 [134, 135]. MUC16, highly glycosylated mucin that is overexpressed in the majority of OCs, has been identified as a surrogate blood biomarker (CA-125) for the diagnosis and prognosis of OCs [136]. CAR-modified MUC16-target T lymphocytes exhibit intense specific cytotoxic activity against MUC16 + OC cells in vitro [137]. Moreover, in a humanized murine model of OC designed to express human mesothelin, mesothelin-targeted IVT CAR T-cells effectively reduced tumor growth [138]. Human FRα-directed IVT mRNA CAR T-cells destroyed human OC cell lines OVCAR3, A187, and SKOV3 in vitro conditions. Furthermore, FRα-targeted mRNA reduced cancer cell proliferation in both localized and diffused mouse models of OC [139]. Finally, the second benefit is that CAR T-cell administration can be accomplished via intraperitoneal injection (IP) instead of intravenous injection (IV). This is attributable to the fact that the peritoneal cavity is the leading site of OC metastases [140].

In addition, explicitly utilizing FRα-specific CAR-NK-92 cells led to the selective lysis of FRα + tumor cells in vitro and a mouse xenograft OC model [141]. Furthermore, in vitro, mesothelin-specific CAR-NK cells were able to eradicate mesothelin-positive OC cells selectively [142]. Other research has recently revealed that co-expression of the chemokine receptors CXCR1 and CAR may provide a unique strategy for improving the therapeutic effectiveness of NK cells in human OC by increasing tumor penetration of effector immune cells [143].

### Lung cancer

Lung cancers are a leading cause of cancer-related mortality, with high resistance to chemotherapies [144], emphasizing the importance of CAR-based therapies for these tumors. A significant proportion of CAR T-cell research in solid tumors is focused on non-small cell lung cancer (NSCLC) [145]. Mesothelin (MSLN), EGFR, PSCA, MUC1, CEA, CD80/CD86, programmed death-ligand 1 (PD-L1), inactive tyrosine-protein kinase transmembrane receptor (ROR1), and HER2 are the most frequently targeted antigens in NSCLC [146]. Also, Kita-Kyushu lung Cancer Antigen-1 (KK-LC-1) is recognized as a potential immunotherapy antigen for lung cancer and some other solid tumor immunotherapy [147]. Other antigens targeted with lung cancer CAR T-cells in clinical trials include Lewis-Y antigen, ganglioside GD2, melanoma-associated antigen (MAGE)-A1, and MAGE-A4 (NCT03198052 and NCT03356808). Mesothelin is one such antigen found in high concentrations in most lung cancers. In patients with early-stage lung adenocarcinoma, mesothelin overexpression is significantly associated with tumor aggressiveness and a lower survival rate [148]. This has resulted in the development of multiple safety methods, which are currently being investigated in multiple clinical trials for mesothelin-targeted CAR T-cell immunotherapy, including NCT01583686. HER2 overexpression was also identified in NSCLC and is shown with a poor prognosis [149]. Currently, numerous clinical trials evaluate the efficacy of HER2-targeted CAR T-cells for HER2-positive solid tumors, including NSCLC (NCT02713984, NCT03740256). CAR T-cell against PD-L1 is also investigated in phase 1 trial of advanced PD-L1-positive NSCLC patients (NCT03330834) (NCT03330834). Wallstabe et al. recently demonstrated that anti-ROR1 CAR T-cells successfully eradicated NSCLC and TNBC cells in three-dimensional tumor models. As a result, anti-ROR1 CAR T-cell therapy provides a new approach to treating NSCLC [150]. Hu et al. developed a CAR T-cell-based method to target lung-specific X receptors (LunX). CARLunX-T-cells exhibited increased toxicity toward NSCLC in vitro and were extensively studied. They also developed a patient-derived xenograft model of lung cancer and demonstrated that survival was incredibly prolonged[151]. Overall, the therapeutic applicability of CAR T-cells in lung cancer treatment is still being researched extensively. However, the continuing progression of CAR T-cell therapy for lung cancer shows great promise.

On the other hand, in animal models of lung cancer, Wang et al. found that inhibiting CD73 increases homing of NKG2D-CAR-NK cells, which target tumor cells expressing NKG2D ligands, and improves antitumor responses [152].

### Colorectal cancer

Colorectal cancer (CRC) refers to colon and rectal epithelial tissue cancers that primarily affect the elderly and have a poor prognosis [153]. Various promising CAR T-cell-based therapeutic targets have been proposed in preclinical models for CRC, including CEA, EGFR, MUC1, NKG2DL, HER2, and CD133 with many therapeutic strategies [154, 155]. Hedge et al. published one of the first human studies utilizing first-generation retrovirally transduced CAR T-cells targeting tumor-associated glycoprotein (TAG)-72 in metastatic CRC. Although these findings indicate that CART72 cells are relatively safe, they show limited persistence in the blood and trafficking to tumor tissue [156]. In addition, the use of CEA-CAR T-cells in various cancers, including CRC, is being investigated in several clinical trials. Zhang et al. found that CEA-targeted CAR T-cells were well-tolerated in CEA + CRC patients, even at high dosages, and some effectiveness was seen in the most treated patients [157]. Katz et al. also discovered that regional intraperitoneal CAR T-cells infusions provided more excellent protection against CEA + mouse colon cancer cells when compared to systemically infused CAR T-cells [158]. On the other hand, the EpCAM is overexpressed in a wide range of tumors, including colorectal cancer. Therefore, Ang et al. reported that utilizing anti-EpCAM-CAR-expressing T-cells for local therapy of peritoneal carcinomatosis in mice is successful [159]. Furthermore, in 2019, Deng et al. reported that NKG2D CAR T-cells had a high killing effect, indicating a promising immunotherapeutic method for human CRC [160]. In a patient-derived colon cancer murine model, the therapeutic efficacy of HER2-specific CAR T-cells on the engrafted tumor was investigated. According to the findings, HER2-specific CAR T-cells could be used to treat colon cancer and show potential in treating other solid tumors [161]. MUC1 CAR T-cell therapy is also proposed for metastatic CRC and has been demonstrated to be safe in humans [162]. Moreover, Magee et al. revealed that GUCY2C mouse model CAR T-cells detected and destroyed human colorectal tumor cells that were endogenously expressing GUCY2C, resulting in long-term survival in an immunodeficient mouse-human xenograft model [163]. According to the studies described, some clinical trials have shown the safety and efficacy of immunotherapy-based CAR T-cells in the treatment of CRC, but not all trials. As a result, CAR T-cell therapy could be a potential therapeutic option for CRC patients in the future.

Besides CAR T-cell, some studies also applied NK CAR products to investigate their efficacy in CRC treatment. Zhang et al. transduced the EpCAM-CAR by using a lentiviral vector on the NK-92 cell line. Anti-EpCAM-CAR-NK-92 cells demonstrated potent antitumor cytotoxicity and cytokine production in EpCAM + colorectal cancer cells. Injecting anti-EpCAM-CAR-NK-92 cells into a CRC xenograft model in conjunction with regorafenib, a protein kinase inhibitor, successfully inhibited tumor development [164].

### Pancreatic cancer

The use of CAR T-cells in the therapy of pancreatic malignancies is still being studied. Although current pancreatic cancer CAR T-cell clinical trials have failed to enhance survival, it has the potential to provide valuable opportunities [165, 166]. A number of clinical trials are now underway, intending to define the usage of this therapeutic technique in the near future. Mesothelin, CD133, PSCA, claudin 18.2, EGFR, CEA, MUC1, and HER2 were among the antigens targeted for therapy in these studies [165, 167, 168]. Mesothelin appears to be the most potential CAR target due to its high expression levels, particularly in mesothelioma, pancreatic, and OC [169]. According to the findings, CAR T-meso destroyed pancreatic cancer cells in vitro and suppressed subcutaneous tumor growth in vivo. In addition, CAR T-meso successfully suppressed lung metastases caused by pancreatic cancer [170, 171]. HER2 has been shown to have a 20–60% higher expression level in pancreatic cancer. CAR against HER2 was designed in a study to inhibit the metastasis and growth of pancreatic xenograft cancer in mice [172]. Another strategy involved targeting pancreatic cancer with CAR T-cells against PSCA and protecting CAR T-cells from the immunosuppressive cytokine IL-4 via the inverted cytokine receptor (4/7 ICR) [173]. Zhang E et al. created a dual CAR-modified T-cell to eliminate AsPC-1 pancreatic cells with high CEA and mesothelin expression [174]. Despite developing multiple pancreatic cancer-targeted CARs, pancreatic cancer heterogeneity and the complex and heterogeneous TME pose significant challenges to successful immunotherapy. To circumvent this obstacle, several researchers have coupled CAR T-cells with additional therapies such as checkpoint inhibitors and oncolytic viruses [175]. Furthermore, a study presented a case of Robo1-CAR-NK cell therapy for Pancreatic ductal adenocarcinoma with liver metastasis [176].

### Glioblastoma

The utilization of CAR T-cells in glioblastoma (GB) has been limited, owing to the lack of well-described tumor-specific antigens that are expressed homogenously and frequently [177]. Cell surface antigens confined to tumor cells are ideal candidates, as they avoid the toxic effects caused by CAR T-cells recognizing normal organ tissue [178]. Multiple GB antigens have been targeted by CAR T-cells in clinical studies, including interleukin-13 receptor alpha 2 (IL-13αR2), epidermal growth factor receptor variant III (EGFRvIII), and Her 2 [178, 179]. EGFRvIII is a constitutively activated tyrosine kinase expressed on the surface of GB and is related to GB development and progression. However, EGFRvIII is not found in normal tissue [180]. Anti-EGFRvIII CARs exhibited promise in preclinical treatment models of GB. Animal survival in a mouse glioma model was directly related to the amount of anti-EGFRvIII CARs injected intracerebrally [181]. EGFR-CAR T-cell therapies are currently being studied in multiple phase 1 and phase 2 trials (NCT01454596, NCT03283631, NCT05024175). Another potential target for CAR T-cell therapies is IL-13Rα2, and its overexpression is associated with a poor prognosis in patients with GB [182]. An IL-13Rα2-specific CAR termed IL-13-zetakine and early results from a second-generation 4-1BB costimulatory IL-13-zetakine CAR showed promising clinical activity [183]. Another target is HER2, which has been extensively studied in breast cancer [184]. Increased HER2 expression in GB is linked to a poor prognosis [185]. HER2 is being studied as a potential target in a number of clinical trials (NCT03389230, NCT03423992). Moreover, to target GB-related surface proteins, some therapeutic trials have used genetically modified cytomegalovirus (CMV)-specific T-cells that express CARs [177]. In this context, Ahmed et al. showed that the administration of autologous HER2-CAR-modified virus-specific T-cells (VSTs) could be correlated with clinical benefit in patients with advanced GB [186]. Mucin 1 (NCT02617134), CD147 (NCT04045847), CD133 (NCT03423992), EphA2 (NCT02575261), and GD2 (NCT02575261) are among the other targets for CAR T-cell therapy in gliomas (NCT02617134).

GB treatment using EGFR-CD28-CD3 CAR-NK cells revealed substantial cytotoxic effects and enhanced murine survival in a tumor-bearing mouse model [187]. Other research has found that further genetic modification of these EGFR-CAR-NK cells with the chemokine receptor CXCR4 improved survival in xenograft mice compared to treatment with EGFR-CAR-NK cells [188]. Other trials have targeted CD73 [152], EGFRvIII [189], and HER2 [190] in GB cell lines, with considerable anti-GB activity and tumor lysis. Multi-targeted CAR-NK cells may be an effective method for treating GB due to the immunosuppressive milieu and the tumor heterogeneity [129].

### Neuroblastoma

Neuroblastoma is a type of brain cancer with a dismal prognosis that primarily affects children. CAR immunotherapy in neuroblastoma has been shown to be safe and feasible in early phase trials, but there are still significant barriers to its efficacy. These include an immunosuppressive TME, difficulties in identifying targets, and limitation of T-cell persistence and efficacy [191]. Despite the preclinical development of CAR T-cells targeting, a variety of neuroblastoma-associated antigens, only those targeting GD2 and L1-CAM (CD171) have progressed to clinical trials [192, 193]. Besides mixed findings from early GD2 CAR clinical trials, this target continues to be a hot topic. Many preclinical studies targeting GD2 are currently underway, as well as several clinical trials on neuroblastoma patients (NCT02765243, NCT02919046, NCT03373097, NCT03294954, and NCT02761915).

CAR-NK cells have also been studied in Neuroblastoma. In a preclinical experiment, ganglioside GD2-specific CAR-NK cells were employed to treat a multi-drug resistant neuroblastoma cell line and a neuroblastoma xenograft model, which demonstrated significant antitumor activity [194]. Moreover, CD276-NK-92 CAR-NK cells could successfully lyse neuroblastoma monolayer and 3D spheroids [195]. Another study suggests that combining redirected NK cells with either PD-1 or PD-L1 blockers or histone deacetylase inhibitors (HDACi) may be more effective against cancer [196].

### Other tumors

There are eleven clinical trials involving CAR cell therapy in melanoma patients. A recent study in humanized mouse models highlights the potential of HER2 as a melanoma target antigen and supports the use of CAR T-cells in patients with highly refractory melanoma [197]. In addition, the use of VEGF-R2-specific CAR T-cells to stop tumor growth by destroying tumor-associated vasculature gave melanoma-bearing mice dramatic survival advantages [198]. Ganglioside GD2 is a well-known antigen in different tumors, including melanoma, and reprogrammed CAR T-cells against GD2 have been shown to lyse primary melanoma cells in vitro and eliminate melanoma in animal models [199]. Furthermore, several clinical trials are investigating the safety and efficacy of CAR T-cells directed against c-MET (NCT03060356), CD20 (NCT03893019), IL13RA2 (NCT04119024), and CD70 (NCT02830724). These clinical trials are phase I or II or single-arm trials which will warrant further investigation if successful.

Hepatocellular carcinoma (HCC) is a lethal type of liver cancer [200]. Some preclinical and clinical evidence suggests that CAR T-cell therapies targeting CEA, MUC-1, and GPC-3 antigens have potent antitumor activity in HCC [201, 202]. The majority of experimental results have come from targeting GPC-3, either with an inducible armored IL-12 construct or by directly eliminating GPC-3 positive HCC cells [203, 204, 205]. Overall, CAR T-cell therapy in HCC still has a long way to go from bench to bedside. CAR-NK cells have been studied in HCC as well. Yu et al. investigated that anti-glypican-3 (GPC3)-CAR-NK cells against HCC cells induced significantly in vitro cytotoxicity and cytokine release, as well as antitumor in vivo effects in HCC xenograft models [206]. In another investigation, NKG2D-DAP10-CD3ζ-CAR-NK cells exhibited a robust antitumor immune response against HCC cell lines of HCC as well as osteosarcoma, pancreatic cancer, and breast cancer [207]. In addition, Wang et al. modified NK-92 cells for expressing the TN chimeric receptor, which combines the transmembrane domains of NKG2D and extracellular of TGF-type II receptor. They discovered that TN-expressing NK-92 cells were significantly resistant to TGF-*β*-induced inhibitory signaling and produced significantly more killing capacity and IFN-*γ* production in vitro against HCC cells [208].

There is limited research on CAR T-cell treatment in gastric cancer [209]. According to a study, developing a CAR T-cell that targets the monoclonal antibody 3H11 produced a favorable response in gastric cancer [210]. Another study revealed that Folate receptor 1 (FOLR1) CAR T-cells could detect and kill FOLR1-positive gastric cancer cells [211]. In the third investigation, the NKG2D ligand was chosen as a target, and the NKG2D-CAR T-cell was found to have cytolytic action against gastric cancer cells [212]. The latest research concerning gastric cancer involved targeting claudin18.2 for its CAR T-cell construction [213].

Several clinical investigations have recently focused on using TAAs as an induction mechanism to stimulate an immune response in patients with prostate cancer (PCa) [214]. Prostate-specific antigen (PSA), prostatic acid phosphatase (PAP), PSCA, T-cell receptor gamma alternate reading frame protein (TARP), transient receptor potential (trp)-p8, and PSMA are potential targets that are preferentially expressed by malignant cells in PCa [215, 216]. In a study, PSCA-DAP12-CAR-NK cells derived from PB-NK and YTS-NK showed substantial tumor lysis against PSCA + PCa cells [217]. In addition, in vitro investigations revealed that anti-PSMA CAR-NK-92 cells secreted a lot of IFN-γ and had significant lytic activity against PCa cells. The administration of PSMA CAR-NK-92 cells to PCa-bearing mice successfully reduced tumor development and enhanced survival [218].

The possible nephrotoxicity of CAR *T*-cell treatment poses a significant risk in patients with renal cancer. Currently, multiple trials are underway in renal cell carcinoma by targeting VEGFR2, CAIX, CCT301-38, CCT301-59, and CD70 [219, 220].

## Limitations and challenges

**Fig. 3:**
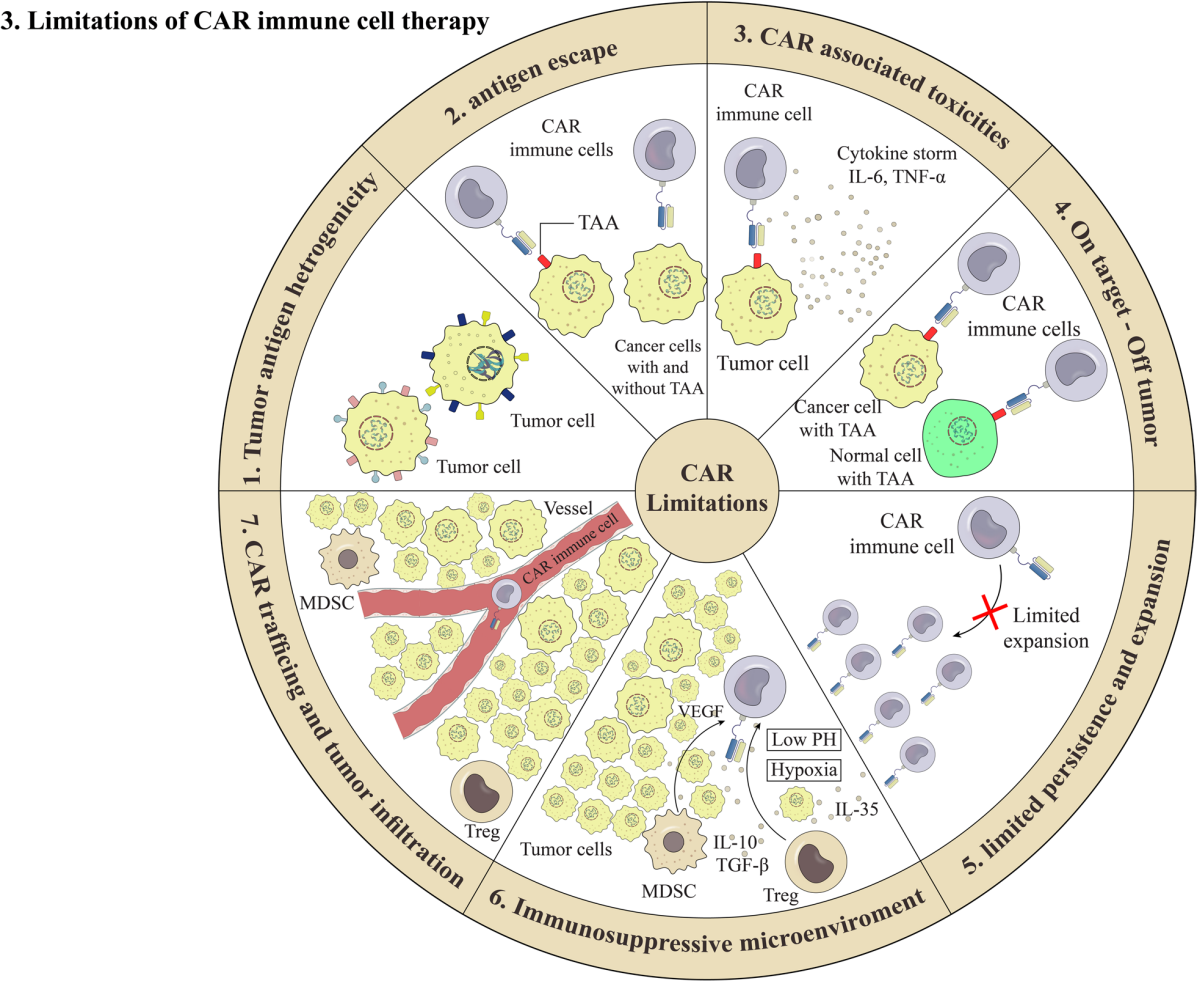
Limitations of chimeric antigen receptor (CAR) T cells. There are several limitations in using CAR-immune cells in tumor therapy, including antigen hetrogenicity, limit proliferation and short retention in tumor site, low trafficking and infiltration of CAR T cells to the tumor site, on target off tumor condition, cytokine release syndrome (CRS), and immunesuppressive TME. Abbreviations: PD-1 (programmed cell death protein 1), TAM (tumor associated macrophage), MDSC (Myeloidderived suppressor cell),T-reg (T regulatory), TAA (tumor associated antigen)

Although CAR immune cell therapy has significant potential as a viable therapeutic method, it has potentially life-threatening side effects and safety problems in clinical applications [221, 222, 223] (Fig. 3). Here, the major limitations of immunotherapy by CAR immune cells are described.

### CAR target selection and tumor antigen heterogeneity

Currently, the most effective way to treat malignancies with CAR immune cells is to identify tumor-specific cell surface antigens [224]. In addition to being tumor-specific, ideal candidate tumor antigens must be expressed homogeneously on the surface of a majority of tumor cells to mediate effective tumor killing. Antigen heterogeneity has been a universal hurdle to effective CAR treatment of a variety of cancers, including CD19-CAR for leukemia and lymphoma [225]. Since the most valuable targets for CAR engineering are TAAs, the diverse expression of TAA by different types of tumor cells is a significant barrier. Furthermore, because malignant cell antigen heterogeneity makes identifying tumor cell-specific antigens challenging, variable levels of antigen expression at distinct tumor locations may impede the activity of CARs at the tumor location [226]. So far, several methods have been utilized to support the target of various TAAs by identified CAR immune cells, including co-expression of multiple CARs on a single immune cell, the transient adjustment of target antigens, programmable CAR expression, exploitation of various CAR immune cells, expression of each chimeric receptor in relation to a particular antigen, and expression of a chimeric receptor including two or more antigen recognition domains, which leads to multiple antigens identifying through the single receptor [227]. On the other hand, targeting cancer stem cells closely related to tumor heterogeneity is one of the methods to eliminate tumor heterogeneity. For example, CD133 is a marker of stem cells that is overexpressed in many solid tumors and is now considered a target tumor marker for CAR T-cells [228].

### Tumor antigen escape

Immune escape is another potential issue [229, 230]. Although CAR T-cells that target a single antigen can achieve high response rates at first, the malignant cells of a significant number of patients administered with these CAR T-cells lose target antigen expression and produce new tumor antigens that the original CAR T-cells may not recognize [7]. Potential solution is targeting the antigens that are expressed on the tumor stroma. The tumor stromal compartment promotes tumor growth by secreting cytokines and growth factors, supplying nutrients, and contributing to tumor-induced immunosuppression [122]. Furthermore, studies which target FAP expressed on cancer-associated fibroblasts or VEGFR-2 expressed on tumor vasculature endothelial cells have shown that tumor stroma is genetically more stable [231, 232]. Furthermore, CAR-expressing NK cells can eradicate malignant cells that lose CAR targeted specific antigen through their innate immune receptors .

### CAR associated toxicities

Unfortunately, as the potency of CARs was increased, high rates of toxicity were observed. The release of a number of inflammatory cytokines by infused T-cells in response to antigen recognition causes cytokine release syndrome (CRS), a systemic inflammatory response with a large increase in the expression levels of TNF-α, C-reactive protein, IL-2, IL-8, IFN-γ, and most importantly, IL-6. Fever, hypotension, anorexia, fatigue, multi-organ dysfunction, and even sudden death can occur due to the cytokine storm [233, 234]. Another common adverse effect of CAR T-cell therapy is nervous system toxicity, including seizures and confusion [235, 236]. Patients with ALL or lymphoma who received the first FDA-approved CAR T-cell therapy, CD19-directed CARs, have had the most extensive characterization of the toxicities underlying CAR T-cell therapy to date [7]. Researchers are modifying T-cell dose escalation and have introduced the prompt use of inhibitors that block the effects of IL-6 (tocilizumab or siltuximab) or TNF-α (Infliximab) to reduce the severity of CRS effects [237, 238]. The identification of CRS biomarkers is another critical issue. Teachey et al. demonstrated that a combination of three cytokines (IFN-γ, soluble IL-1 receptor agonist, and glycoprotein subunit 130) accurately predicted CRS one month after anti-CD19 CAR T-cell infusion for ALL [239]. On the other hand, early promising CAR-NK studies indicate significant lack of CRS or neurotoxicity. So that in the study by Liu et al., administration of anti-CD19 CAR-NK cells was not associated with the development of CRS, neurotoxicity, or GVHD, and there was no increase in inflammatory cytokines, including interleukin-6, above baseline [240].

### On-target off-tumor effects

The activation of CAR T-cells by targeting antigen within healthy tissues is causing “on-target, off-tumor” toxicity, which is currently a major concern. This occurs because many TAAs are not tumor-specific and are also expressed at varying levels in normal tissues [241, 242]. On-target toxicity prevention requires the proper selection of antigens with more restricted expression [243]. Other strategies are infusing CAR T-cells with only temporary CAR expression (the expression level decreases with cell division, and transcription gradually becomes diluted) and targeting tumor-specific post-translational modifications such as overexpressed truncated O-glycans in solid tumors [243, 244, 245]. However, the risk of on-target/off-tumor toxicity to normal tissues by CAR-NK cells is low because of the short lifespan of CAR-NK cells in circulation. Moreover, previous research discovered that optimizing the affinity of tumor antigen binding resulted in a CAR with improved selectivity to high tumor antigen density cells and a decreased risk of on-target off-tumor effects [246, 247]. This enhanced therapeutic index was primarily achieved by tuning down the affinity of the scFv antibody for its cognate target antigen sequence. This process seemed to have a greater impact when membrane antigen expression was low. Affinity tuning with purified antigen sequences ignores the spatial arrangement of antigen on the cell membrane. As a risk-mitigation strategy, the approach could increase off-target risk [248].

### Limited expansion and persistence

A massive amount of CAR immune cells is required for achieving optimal clinical-scale responses. As the number of immune cells from a single donor is insufficient for treatment, immune cell expansion and activation are critical [249]. The limitations of the strong in vivo persistence and expansion of CAR T-cells, particularly CAR-NK cells, are a major barrier to therapeutic efficacy, while this feature may be desirable in terms of safety [250, 251]. Therefore, CARs can be genetically modified to produce cytokines or add cytokines/cytokine receptors to improve in vivo expansion and persistence while avoiding systemic toxicity [122]. Furthermore, CAR T-cell function may be improved by combining immunotherapy with checkpoint blockade [252]. In addition, exogenous cytokines may be used to expand and activate NK cells [39]. Another way to boost NK cell persistence is to give them a memory-like phenotype by pre-activating them with a cytokine cocktail (IL-12, IL-15, and IL-18) for a brief period to induce differentiation into cytokine-induced memory-like NK cells [253]. Moreover, knocking out the cytokine-inducible sh2-containing protein (CISH) gene, a negative regulator of IL-15 signaling, has been shown to improve NK cell metabolism, persistence, cytotoxicity, and CAR-NK functionality [254]. Notably, in an AML xenograft model, CISH knockout NK cells mediated improved disease control [255]. Besides, researchers optimized the structure of CAR and introduced the IL-15 transgene (iC9/CAR.19/IL15) to increase the persistence time of CAR-NK cells in vivo and reduce treatment-related side effects. In xenogeneic mouse models of lymphoma, such a novel design demonstrated remarkable survival [256].

### Immunosuppressive microenvironment

Studies demonstrated that rapid loss of CAR immune cell function limits its therapeutic role in immunosuppressive TME [4]. Different cell types (Tregs, MDSCs, and TAMs) can infiltrate solid tumors and support tumor growth, angiogenesis, and metastasis. In addition to tumor cells, these cells produce growth factors, chemokines, and local cytokines, such as IL-10, VEGF, TGF-β, and IL-4 which stimulate tumor growth and proliferation. Antitumor immunity is also reduced by immune checkpoint molecules like CTLA-4, TIM3, PD-L1 and PD-1 [257, 258], and their combinational blockade has shown great promises for cancer treatment [259]. Furthermore, solid tumors frequently show metabolic aberrations that can affect immune cell biology, such as increased metabolism and subsequent depletion of amino acids (e.g., arginine) that are important for immune cell function, also hypoxia and extracellular matrix acidification due to insufficient vascular supply that is hostile to T-cell survival [260]. As a result, various approaches have been employed to arm CAR immune cells against tumor immunosuppressive microenvironment, such as enhancing CAR immune cells performance by altering metabolic profiles to increase cell activity in hostile environments or combining CARs with immune checkpoint inhibitors like monoclonal antibodies. Remarkably, in some types of cancer, including multiple myeloma, mesenchymal stromal cells in the tumor milieu have been found to be involved in cancer resistance to CAR T-cell therapies by upregulating survivin [101], which has been shown to serve as an anti-apoptotic factor in a variety of pathological conditions [261, 262]. As well, immunomodulatory cytokines like IL-12, which increase the immune response via NK cells and T-cells, can also be used to improve CAR immune cell therapy [263, 264]. Transforming growth factor-beta (TGF-*β*) CAR T-cells also can help with antitumor immune functions by shielding neighboring immune cells from TGF-β 's immunosuppressive effects [265]. Moreover, dominant-negative receptors like TGF-βRII that act as a sink for immunosuppressive cytokines has been developed and thus improve CAR T-cell function in the TME [266]. Furthermore, silencing an intracellular immune checkpoint in NK cells (CRISPR/Cas9-mediated CISH knockout (CISH KO)) improves their metabolic fitness in the TME and leads to enhanced functions [267].

### Limited CAR trafficking and tumor infiltration

Another significant barrier for CAR immune cells in solid tumors is the need for immune cells to extravasate from the bloodstream into solid tissues to facilitate antitumor activity. During these processes, CAR immune cells must attach to endothelial cells and initiate chemokine-chemokine receptor interactions in order to extravasate into tumor areas [260, 268]. On the other hand, CAR T-cell therapy’s efficacy in B-cell malignancies is probably since target B-cells are easily accessible to CAR T-cells and express a variety of costimulatory receptor ligands that can increase CAR T-cell function [269]. Solid tumor CAR immune cell therapy is limited compared to hematological malignancies due to dysregulation in cytokine secretion by tumor cells and low chemokine receptor expression on CAR immune cells, as well as the existence of a thick fibrotic matrix in solid tumors, which limits CAR’s capacity to migrate and invade cancerous cells [270, 271]. One strategy for overcoming these restrictions is to use localized delivery rather than systemic administration [272]. Another strategy for cancer immunotherapy is to target the chemokine system (which includes chemokines and their receptors) [273].

## CAR-modified macrophages

Due to the challenges associated with CAR T-cell and NK cell therapies, CAR macrophages (CAR-M) have recently emerged as an alternative therapeutic intervention. CAR-M cells, same as CAR T- and CAR-NK cells, are composed of extracellular signaling domains that recognize specific TAs, transmembrane regions, and intracellular domains [274]. Currently, research into the extracellular signal domain has identified several common tumor targets, such as HER2 (NCT04660929). CAR-M research focuses on activating and enhancing the phagocytic effect through various intracellular domains [274].

CAR macrophages have unique benefits over CAR T-cells in overcoming some major barriers in solid tumors. While T-cells are unable to enter the tumor microenvironment (TME) due to physical barriers formed by the matrix surrounding the tumor cells, macrophages can immerse significantly in the TME [275]. TAM plays a key role in immune suppression, tumor invasion and metastasis [276]. CAR-M can decrease TAM ratios and change TAM cellular phenotypes, which has positive effects on tumor treatment. Besides tumor cell phagocytosis, CAR-M can promote antigen presentation and enhance the cytotoxic effects of T-cells. Furthermore, CAR-M has a shorter time in circulation and less non-tumor toxicity than CAR T [277]. Moreover, macrophages can sense hypoxia and its metabolites, such as low pH, and migrate into the TME [278].

Although CAR-M has the potential to be potent cancer immunotherapy, however, CAR-M is still in its early stages, with only two clinical trials being initiated (NCT05007379, NCT04660929) launched and no results reported. As a result, many limitations have not yet been uncovered. Significant efforts are underway to optimize CAR macrophage structure, manufacturing, storage, tumor infiltration, and retention to cytotoxicity. Repeated dosing may be required to keep CAR macrophage levels high enough for active cancer surveillance [278].

## Conclusion

Based on collective evidence, CAR immune cell-based cancer therapy has become a compelling and advanced research area for targeting malignant cells. Despite significant challenges, several investigations are being conducted to address these constraints. The further success of CAR immune cell therapy will strongly depend on the development of CAR constructs optimally suited for individual applications, the development of toxicity reduction strategies, identification of the most appropriate cell types to express CARs and the possibility to safely and completely remove CAR cells upon achievement of complete remission of the respective disease. Once resolved, CAR cell therapy may become an effective, affordable, and safe treatment for malignancies.

## Data Availability

Not applicable.

## Abbreviations

TRUCKs: T-cells redirected for universal cytokine killing
PSCA: Prostate stem-cell antigen
HSCT: Allo-hematopoietic stem-cell transplantation
GVHD: Graft-versus-host disease
PBMCs: Peripheral blood mononuclear cells
HPCs: Hematopoietic progenitor cells
iPSCs: Induced pluripotent stem cells
GM-CSF: Granulocyte–macrophage colony-stimulating factor
axi-cel: Axicabtagene
AML: Acute myeloid leukemia
CLL: Chronic lymphocytic leukemia
BCMA: B-cell maturation antigen
TNF: Tumor necrosis factor
EpCAM: Epithelial cell adhesion molecule
EGFR: Epidermal growth factor receptor
TNBC: Triple-negative breast cancer
VEGF-R2: Vascular endothelial growth factor receptor 2
CRC: Colorectal cancer
TAG: Tumor-associated glycoprotein
GB: Glioblastoma
CRS: Cytokine release syndrome
CAR-M: CAR macrophages
TME: Tumor microenvironment
TGF-β: Transforming growth factor-beta
